# Inhibition of *let-7c* Regulates Cardiac Regeneration after Cryoinjury in Adult Zebrafish

**DOI:** 10.3390/jcdd6020016

**Published:** 2019-04-04

**Authors:** Suneeta Narumanchi, Karri Kalervo, Sanni Perttunen, Hong Wang, Katariina Immonen, Riikka Kosonen, Mika Laine, Heikki Ruskoaho, Ilkka Tikkanen, Päivi Lakkisto, Jere Paavola

**Affiliations:** 1 Unit of Cardiovascular Research, Minerva Institute for Medical Research, Biomedicum Helsinki, 00290 Helsinki, Finland; suneeta.narumanchi@helsinki.fi (S.N.); karri.kalervo@helsinki.fi (K.K.); sanni.perttunen@minervainstitute.fi (S.P.); hong.wang@helsinki.fi (H.W.); laura.k.immonen@jyu.fi (K.I.); riikka.kosonen@minervainstitute.fi (R.K.); mika.laine@hus.fi (M.L.); ilkka.tikkanen@helsinki.fi (I.T.); paivi.lakkisto@helsinki.fi (P.L.); 2 Department of Surgery, South Karelia Central Hospital, 53130 Lappeenranta, Finland; 3 Heart and Lung Center, University of Helsinki and Helsinki University Hospital, 00029 Helsinki, Finland; 4 Drug Research Programme, Division of Pharmacology and Pharmacotherapy, University of Helsinki, 00014 Helsinki, Finland; heikki.ruskoaho@helsinki.fi; 5 Abdominal Center, Nephrology, University of Helsinki and Helsinki University Hospital, 00029 Helsinki, Finland; 6 Clinical Chemistry and Hematology, University of Helsinki and Helsinki University Hospital, 00014 Helsinki, Finland; 7 Clinical Neurosciences, Neurology, University of Helsinki and Jorvi Hospital of Helsinki University Hospital, 02740 Espoo, Finland

**Keywords:** *let-7c*, miRNAs, epicardial cells, cardiac regeneration, proliferating cardiomyocytes, fibrin

## Abstract

The *let-7c* family of micro-RNAs (miRNAs) is expressed during embryonic development and plays an important role in cell differentiation. We have investigated the role of *let-7c* in heart regeneration after injury in adult zebrafish. *let-7c* antagomir or scramble injections were given at one day after cryoinjury (1 dpi). Tissue samples were collected at 7 dpi, 14 dpi and 28 dpi and cardiac function was assessed before cryoinjury, 1 dpi, 7 dpi, 14 dpi and 28 dpi. Inhibition of *let-7c* increased the rate of fibrinolysis, increased the number of proliferating cell nuclear antigen (PCNA) positive cardiomyocytes at 7 dpi and increased the expression of the epicardial marker *raldh2* at 7 dpi. Additionally, cardiac function measured with echocardiography recovered slightly more rapidly after inhibition of *let-7c*. These results reveal a beneficial role of *let-7c* inhibition in adult zebrafish heart regeneration.

## 1. Introduction

Cardiovascular diseases are the leading cause of death globally [1]. Cardiac remodeling after myocardial infarction in mammals leads to scar formation and hypertrophy [2,3]. Adult zebrafish hearts regenerate completely without scar formation after cryoinjury [4,5], making zebrafish a useful animal model for studying cardiac regeneration [6,7,8]. Cell-lineage-tracing studies with zebrafish have shown complete regeneration after amputation through proliferation of pre-existing cardiomyocytes [9,10,11,12], unlike in mammals [3]. Epicardial-derived cells undergo epithelial-mesenchymal transition to form coronary smooth muscle cells and fibroblasts [11,13,14]. The epicardium plays a crucial role in zebrafish heart regeneration [13,14], as epicardial markers such as *tbx18, tcf21* and *raldh2* are re-expressed after cryoinjury, suggesting that tissue injury reactivates developmental genes [15,16]. We used the cryoinjury method to simulate myocardial infarction. Compared to amputation, cryoinjury more closely mimics the physiological responses associated with myocardial infarction, including cell death, inflammation and scarring [4,5,17]. Cell death and inflammation in the infarct area is followed by simultaneous proliferation of endocardium, epicardium and cardiomyocytes [4,5]. During the first few weeks, fibrin, collagen and other extracellular matrix proteins accumulate in the injury area [4,5,18,19]. The adult zebrafish heart completely regenerates and scar tissue is replaced by functional cardiac tissue, in approximately two months following cryoinjury [11].

Novel therapies are required for improving cardiac remodeling and function after injury. MiRNAs are short, noncoding RNA molecules that act as negative regulators of gene expression by inhibiting mRNA translation or promoting mRNA degradation. They were first studied in model organisms, such as *Caenorhabditis elegans* and *Drosophila* [20,21]. MiRNAs are involved in cell proliferation, apoptosis, hematopoiesis and oncogenesis [22]. The *let-7* family of miRNAs is expressed during embryonic development [23]. *let-7c* is ubiquitously expressed in the whole body in mice [24]. *let-7c* is required for cell growth and differentiation, and its sequence and function is highly conserved [25]. *let-7c* is upregulated in human cardiac maturation in vitro. Overexpression of the *let-7* family of miRNAs matures human embryonic stem cell-derived cardiomyocytes, increases cell size, sarcomere length and contraction [26,27]. *let-7* miRNAs are upregulated in mouse and human heart disease samples [28,29]. Inhibition of *let-7c* improves cardiac remodeling and function after infarction in mice [30]. Another study in mice reported increased recruitment of epicardial cells after *let-7c* inhibition [23]. *let-7a/b* overexpression reduces collagen I expression, cardiac fibroblast proliferation and migration in primary cardiac fibroblast cultures from rats [31].

The aim of our study is to elucidate the role of *let-7c* in cardiac regeneration after cryoinjury in adult zebrafish.

## 2. Materials and Methods

### 2.1. Zebrafish

Zebrafish aged 6–18 months were obtained from the breeding line of a Turku strain maintained in the zebrafish core facility at the University of Helsinki [32]. They were housed at 28 °C with a 14:10 h light:dark cycle. The permit for zebrafish experiments was obtained from the Regional State Administrative Agency for Southern Finland in agreement with the ethical guidelines of the European convention (ESAVI/2988/04.10.07/2014 and ESAVI/4131/04.10.07/2017).

### 2.2. Cryoinjury

Two hundred and forty wild type adult zebrafish were anesthetized with 0.03% tricaine and a small incision was made on the ventral side of the fish for direct access to the heart. Then, a metal probe cooled in liquid nitrogen was applied to the ventricle, resulting in death of approximately 20% of ventricular cardiomyocytes. Sham operations were performed similarly with a metal probe at room temperature.

### 2.3. let-7c Inhibition

To study cardiac function and regeneration after inhibition of *let-7c*, the single stranded custom *let-7c* antagomir (5′-TACAACCTACTACCTC) was synthesized using locked-nucleic-acid-modified (LNA) technology (Exiqon, Vedbaek, Denmark). A custom scramble sequence LNA antagomir (5′-ACGTCTATACGCCCA) was used as control (Exiqon, Vedbaek, Denmark). Initially two different doses (20 mg/kg and 100 mg/kg) were tested and 20 mg/kg was chosen, as it was sufficient for inhibition of *let-7c*. Lyophilized *let-7c* antagomir or custom scramble was resuspended in phosphate buffered saline (PBS) as 10 mg/mL and stored at −20 °C until use. *let-7c* antagomir or scramble was administered as intra-peritoneal injections (20 mg/kg) at 1 dpi.

### 2.4. Echocardiography

Echocardiography was performed with Vevo 2100 and 50 MHz ultrasound probe (FUJIFILM VisualSonics, Toronto, ON, Canada). The fish were first anesthetized in 0.03% tricaine for one minute, followed by anesthesia in 0.015% tricaine during echocardiography. B-mode videos were recorded before cryoinjury, 1 dpi, 7 dpi, 14 dpi, 21 dpi and 28 dpi. Recorded B-mode videos were analyzed with Visual Sonics software as described previously [33,34]. Briefly, during five consecutive heartbeats, systolic and diastolic ventricular volumes were determined and the epicardial edge was manually marked; diastolic and systolic lengths of the apical image long axis (L) and its diastolic and systolic area (A) are measured (Figure 6A). Diastolic and systolic ventricular volumes (V) are then calculated using the formula:

(1) $$V=\frac{8A^{2}}{3\pi L}$$

(2) $$\mathrm{FVS}=100*\frac{\mathrm{diastolic}\;\mathrm{volume}-\mathrm{systolic}\;\mathrm{volume}}{\mathrm{diastolic}\;\mathrm{volume}}$$

### 2.5. RNA Isolation and Quantitative Real-Time PCR (qPCR)

Heart, kidney and liver tissues were dissected at 7 dpi, 14 dpi and 28 dpi time-points, snap-frozen in liquid nitrogen and stored at −80 °C. RNA was extracted with miRNeasy mini Kit (Qiagen, Hilden, Germany) or RNeasy mini kit (Qiagen, Hilden, Germany), following the manufacturer’s instructions. One hundred and fifty ng of total RNA was converted to cDNA with miScript II RT kit (Qiagen, Hilden, Germany) or Superscript Vilo cDNA synthesis kit (Invitrogen, Carlsbad, California, United States). The *let-7c* gene was amplified with miScript SYBR Green PCR kit (Qiagen, Hilden, Germany) and Hs_*let-7c*_1 miScript Primer Assay (Qiagen, Hilden, Germany) using Hs_*rnu6*-2_11 miScript primer assay (Qiagen, Hilden, Germany) as internal control. Other genes, plasminogen activator inhibitor-1 (*pai-1*), retinaldehyde dehydrogenase 2 (*raldh2*), collagen 12a1a (*col12a1a*) and periostin (*postna*) were amplified with LightCycler 480 SYBR Green I Master (Roche, Basel, Switzerland) using *rps3* as internal control; all primer sequences are listed in Table 1. LightCycler480 II (Roche, Basel, Switzerland) was used for real-time PCR and the expression levels were quantified by the ΔΔCT method.

### 2.6. 5-Bromo-2-Deoxyuridine (BRDU) Baths

BRDU was used to identify the formation of new cardiomyocytes after cryoinjury. Zebrafish were kept in BRDU baths (50 μg/mL) 6 h/day, 5 times a week during the experimental period.

### 2.7. Histology

Acid fuchsin orange G-stain (AFOG) was used for measuring infarct size and fibrosis as described previously [16]. Briefly, paraffin sections were dewaxed and post-fixed in Bouin’s solution for 60 min at 60 °C in an incubator, stained with Weigert’s iron hematoxylin and AFOG-solution. The infarct region was measured using Image J 1.43u software (National Institutes of Health, Bethesda, MD, USA) and the percentage of the infarct size relative to the ventricle size was calculated. Fibrin and collagen percentages were also measured similarly from the infarct region.

### 2.8. Immunohistochemistry

Formation of new cardiomyocytes was analyzed using BRDU labelling or PCNA staining. Dissected adult zebrafish hearts were fixed in 10% formalin overnight, dehydrated in ethanol and embedded in paraffin. Sections (5 μm) were deparaffinized, rehydrated and antigens retrieved by heating in citrate buffer, pH 6.0. Sections were incubated with anti-mouse myosin heavy chain (MHC) (Merck Millipore, Billerica, MA, USA), anti-rat BRDU (Thermofisher, Waltham, MA, USA), anti-rabbit mef-2 (Santa Cruz, California, USA) and anti-mouse PCNA (Cell Signalling, Danvers, MA, USA) at 4 °C overnight, washed with PBS followed by incubation with AlexaFluor-488-conjugated goat anti-mouse, AlexaFluor-594-conjugated goat anti-rabbit and AlexaFluor-594-conjugated goat anti-rat secondary antibodies at room temperature for 1 h. DAPI (4′,6-Diamidino-2-Phenylindole, Dihydrochloride) (Molecular Probes, Eugene, OR, USA) was used for labelling nuclei. Samples were imaged with Leica DM 4500B fluorescence microscope (Leica microsystems, Wetzlar, Germany) and Image J 1.43u software was used for counting proliferating cardiomyocytes. PCNA positive cardiomyocytes were counted from the whole ventricle, PCNA positive and mef-2 positive cells were considered new cardiomyocytes. BRDU positive cardiomyocytes were counted from border area of infarct, BRDU positive and MHC positive cells were considered new cardiomyocytes. PCNA positive or BRDU positive cardiomyocytes were expressed as positive cardiomyocytes/mm^2^, as described previously [36].

### 2.9. Statistical Analysis

Data were analyzed with nonparametric Kruskall–Wallis test followed by Mann–Whitney U test or two-tailed Student’s *t*-test. A value of *p* < 0.05 was considered statistically significant. Results are shown as mean **±** SEM, * *p* ≤ 0.05, ** *p* ≤ 0.005, *** *p* ≤ 0.0005.

## 3. Results

### 3.1. let-7c Expression after Cryoinjury

**Figure 1:**
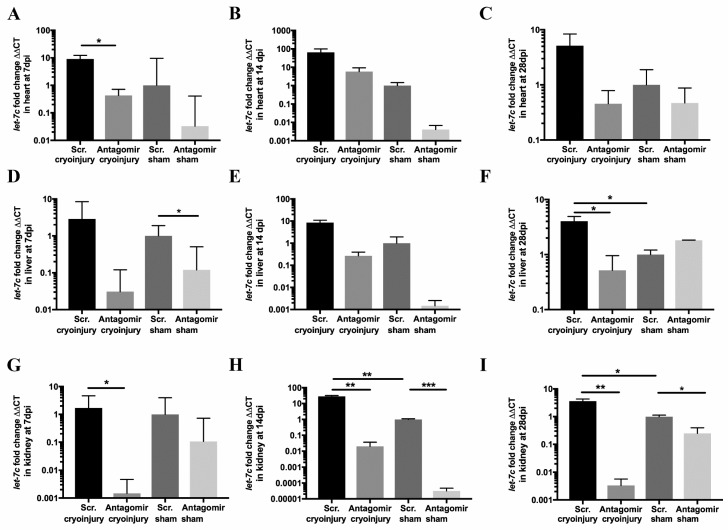
*let-7c* expression in the heart (**A**–**C**), liver (**D**–**F**), and kidney (**G**–**I**). The internal control gene Hs_*rnu6*-2_11 used for normalizing the expression levels by the ΔΔCT method. Expression levels are normalized to scramble (Scr.) sham values in each panel (*n* = 5 in cryoinjured hearts at all time-points, *n* = 3 in all other groups). * *p* ≤ 0.05, ** *p* ≤ 0.005, *** *p* ≤ 0.0005.

To elucidate the efficacy of the *let-7c* antagomir, we quantified *let-7c* expression by qPCR at 7 dpi, 14 dpi and 28 dpi post cryoinjury or sham operation. Scramble-injected cryoinjury or sham-operated fish were used as controls. In the cryoinjured hearts at 7 dpi, *let-7c* antagomir treatment decreased relative *let-7c* expression (0.43 ± 0.28, *p* = 0.03) compared to scramble-treatment (8.10 ± 3.25) (Figure 1A). At 7 dpi relative *let-7c* expression decreased in the liver in sham-operated *let-7c* antagomir-treated fish (0.12 ± 0.38, *p* = 0.02) compared to scramble-treated fish (1 ± 0.91) (Figure 1D). At 28 dpi in the liver, cryoinjury increased *let-7c* expression (*p* = 0.03), and in cryoinjured fish, *let-7c* expression decreased in *let-7c* antagomir fish (0.52 ± 0.43, *p* = 0.023) compared to scramble-treated fish (12.12 ± 0.89). In the kidney in cryoinjured fish, relative *let-7c* expression decreased in antagomir-treated fish at 7 dpi (0.001 ± 0.003, *p* = 0.02) and at 14 dpi (0.02 ± 0.02, *p* = 0.002) compared to scramble-treated fish (1.70 ± 2.95 and 28.27 ± 3.70, respectively) (Figure 1G,H). In the kidney at 14 dpi cryoinjury increased *let-7c* expression (*p* = 0.002). Downregulation of *let-7c* expression was observed at 14 dpi in kidney samples of sham-operated antagomir fish (3.16 × 10^−5^ ± 1.53 × 10^−5^, *p* = 0.0004) compared to scramble treatment (1 ± 0.09) (Figure 1H). In the kidney at 28 dpi cryoinjury increased *let-7c* expression (*p* = 0.02). At 28 dpi *let-7c* expression was downregulated in kidney samples in the cryoinjury *let-7c* antagomir-treated fish (0.003 ± 0.002, *p* = 0.006) compared to scramble-treated fish (3.62 ± 0.70), as well as in the sham-operated fish (antagomir 0.25 ± 0.15 vs. scramble 1 ± 0.13, *p* = 0.02) (Figure 1I).

### 3.2. Fibrin and Collagen Deposition after Cryoinjury

**Figure 2:**
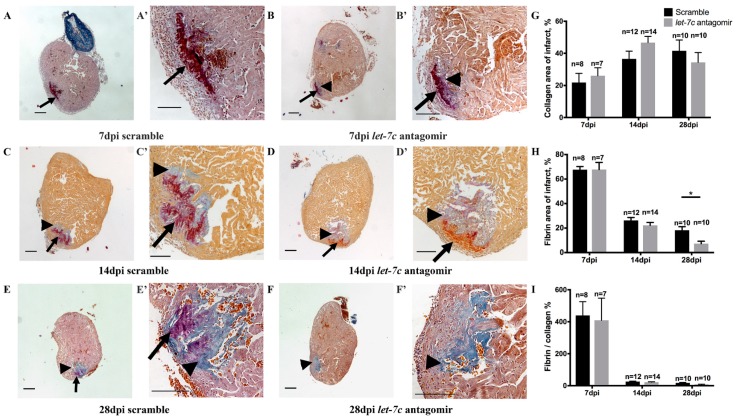
Collagen and fibrin quantified with AFOG staining at 7 dpi, 14 dpi and 28 dpi. Intact myocardium is stained in orange, fibrin in red and collagen in blue (**A**–**F**); higher magnifications of the panels on the left (**A’**–**F’**). Arrows point to fibrin and arrowheads to collagen (**A**–**F**; **A’**–**F’**). Collagen (**G**), fibrin (**H**) and fibrin/collagen% (I) (*n* = 7 for 7 dpi antagomir, *n* = 8 for 7 dpi scramble, *n* = 14 for 14 dpi antagomir, *n* = 12 for 14 dpi scramble, *n* = 10 for 28 dpi antagomir and scramble). Scale bar: 200 μm. * *p* ≤ 0.05.

Fibrosis after cryoinjury was quantified using AFOG staining. The fibrin amount was lower in *let-7c* antagomir fish in the infarct area (7.25 ± 1.97%, *p* = 0.008) at 28 dpi compared to scramble-treated fish (18.21 ± 2.87%) (Figure 2H). Collagen deposition and fibrin/collagen ratio were similar between the groups (Figure 2G,I).

### 3.3. Expression of pai-1

**Figure 3:**
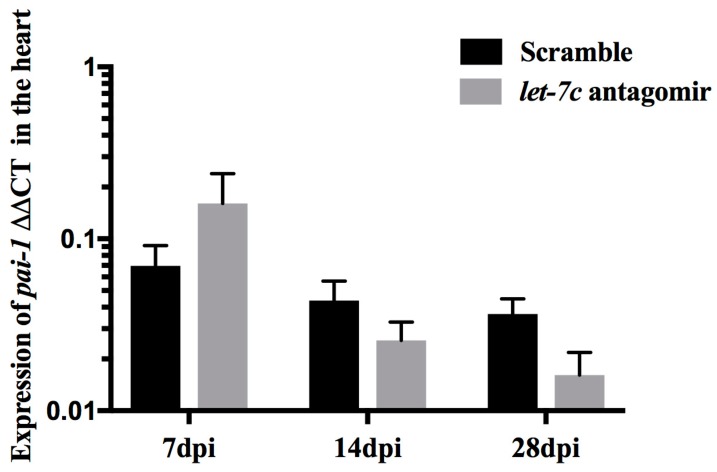
*pai-1* expression in the heart quantified by qPCR at 7 dpi, 14 dpi and 28 dpi. The internal control gene *rps3* used for normalizing the expression levels by the ΔΔCT method. (*n* = 6 for 7 dpi antagomir and scramble, *n* = 5 for 14 dpi antagomir and scramble, *n* = 4 for 28 dpi antagomir and *n* = 5 for 28 dpi scramble).

*pai-1* expression was quantified to study the increased rate of fibrinolysis observed in the let-7c antagomir fish. *pai-1* expression remained similar. However, there was a trend for lower relative *pai-1* expression in the heart at 28 dpi in *let-7c* antagomir fish (0.02 ± 0.01, *p* = 0.10) compared to scramble fish (0.04 ± 0.01) (Figure 3).

### 3.4. Proliferating Cardiomyocytes

**Figure 4:**
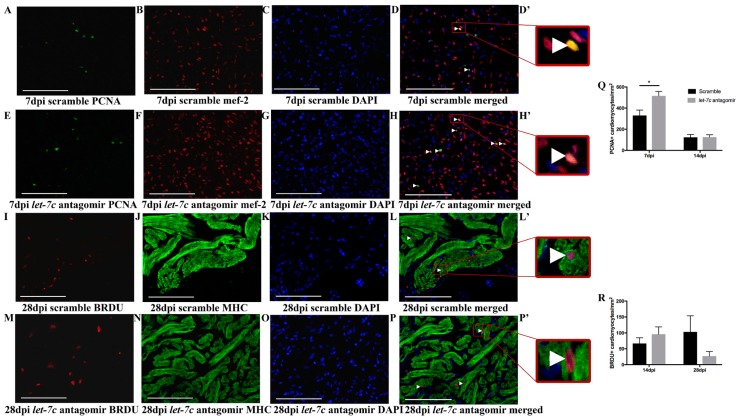
Quantification of proliferating cardiomyocytes in cryoinjured hearts**.** Representative images at 7 dpi of PCNA, mef-2, DAPI, merged image and inset of merged image in scramble (**A**–**D**,**D’**) and in *let-7c* antagomir hearts (**E**–**H**,**H’**). Representative images at 28 dpi of BRDU, MHC, DAPI, merged image and inset of merged image in scramble (**I**–**L**,**L’**) and in *let-7c* antagomir hearts (**M**–**P**,**P’**). Positive cardiomyocytes marked with arrowheads. In PCNA images (A–H’) mef-2 marks cardiomyocytes in red, PCNA positive cells marked in green and DAPI nuclei marked in blue. In BRDU images (**I**–**P’**), MHC marks myocardium in green, BRDU positive cells marked in red and DAPI nuclei marked in blue. Insets are magnified images of merged image (**D’**,**H’**,**L’**,**P’**). Newly formed cardiomyocytes are quantified by PCNA staining at 7 dpi and 14 dpi (**Q**) and by BRDU labeling at 14 dpi and 28 dpi (**R**). (PCNA: *n* = 3 for 7 dpi antagomir and scramble, *n* = 8 for 14 dpi antagomir and scramble. BRDU: *n* = 10 for 14 dpi antagomir, *n* = 9 for 14 dpi scramble, *n* = 8 for 28 dpi antagomir and *n* = 6 for 28 dpi scramble). Scale bar: 100 μm. * *p* ≤ 0.05.

Proliferating cardiomyocytes in cryoinjured hearts were counted at 7 dpi and 14 dpi with PCNA staining (Figure 4A–H’,Q), and at 14 dpi and 28 dpi with BRDU staining (Figure 4I–P’,R). At 7 dpi the number of proliferating cardiomyocytes was increased with PCNA staining in *let-7c* antagomir fish (515.44 ± 42.15 cells/mm^2^) compared to scramble-treated fish (330.63 ± 50.83 cells/mm^2^) (Figure 4Q). BRDU staining showed similar levels of proliferating cardiomyocytes in *let-7c* antagomir and scramble fish at 14 dpi and 28 dpi (Figure 4R).

### 3.5. Epicardial Activation through raldh2 Expression

**Figure 5:**
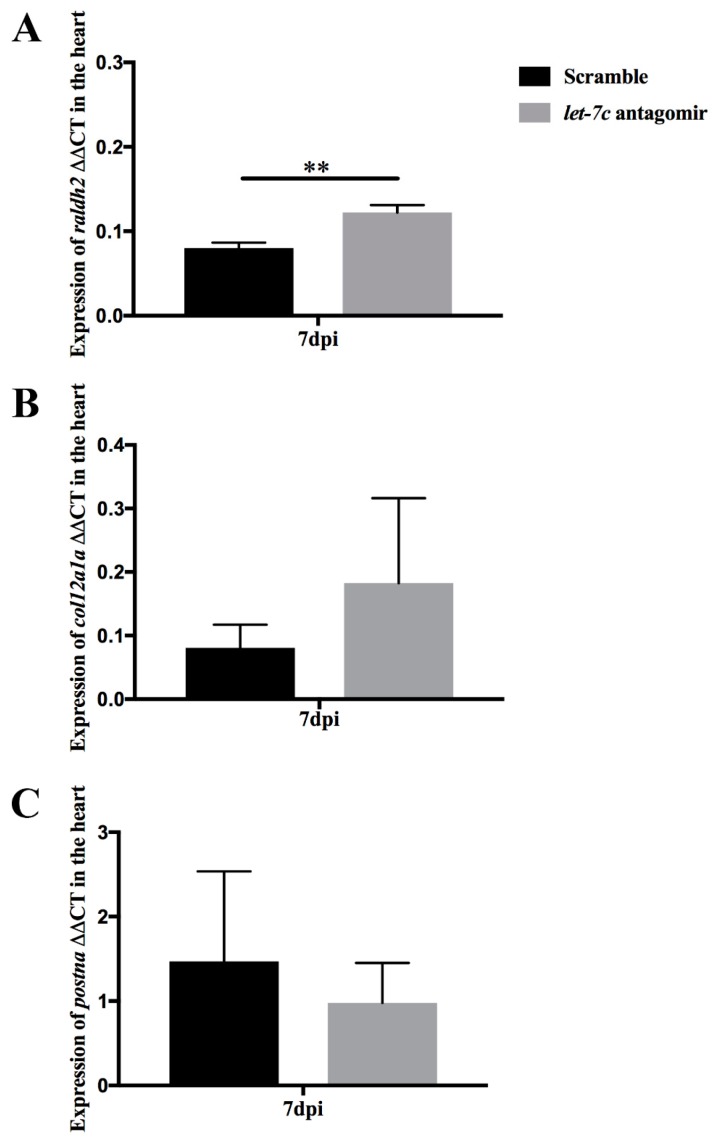
Marker genes of activated epicardial cells ((**A**); *raldh2*) and fibroblasts ((**B**) *col12a1a*, (**C**); *postna*) quantified with qPCR from hearts at 7 dpi. The internal control gene *rps3* used for normalizing the expression levels by the ΔΔCT method. (*n* = 6 in all groups). ** *p* ≤ 0.005.

Inhibition of *let-7c* increased recruitment of epicardial cells in mice [23]. In the cryoinjured heart at 7 dpi *let-7c* antagomir fish showed increased relative *raldh2* expression (0.12 ± 0.01, *p* = 0.004) compared to scramble-injected fish (0.08 ± 0.01) (Figure 5A). *let-7c* inhibition did not cause significant changes in *col12a1a* and *postna* expression in cardiac fibroblasts and activated fibroblasts, respectively (Figure 5B,C).

### 3.6. Echocardiography

**Figure 6:**
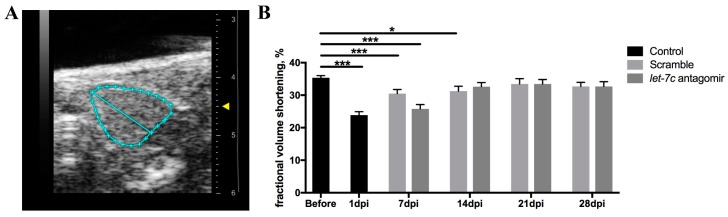
Echocardiography to assess cardiac function. The epicardial border is marked (**A**). FVS measured from recorded B-mode videos before cryoinjury, and at 1 dpi, 7 dpi, 14 dpi, 21 dpi, and 28 dpi (**B**). (*n* = 60 for before, *n* = 18 for 1 dpi, *n* = 29 for 7 dpi antagomir, *n* = 30 for 7 dpi scramble, *n* = 28 for 14 dpi antagomir, *n* = 29 for 14 dpi scramble, *n* = 16 for 21 dpi antagomir, *n* = 18 for 21 dpi scramble, *n* = 16 for 28 dpi antagomir and *n* = 17 for 28 dpi scramble). * *p* ≤ 0.05, *** *p* ≤ 0.0005.

Echocardiography was used to assess cardiac function before and after cryoinjury. Fractional volume shortening (FVS) was measured from recorded B-mode videos (Figure 6A,B). FVS was similar in antagomir and scramble fish at all time-points. However, FVS returned to pre-cryoinjury levels (35.61% ± 0.96%) at 14 dpi (32.61% ± 1.28%, *p* = 0.069) in the antagomir fish, and at 21 dpi (33.46% ± 1.63%, *p* = 0.229) in the scramble fish.

## 4. Discussion

Our study reveals that inhibition of *let-7c* results in an increased rate of fibrinolysis, increased number of proliferating cardiomyocytes, higher expression of the epicardial cell marker *raldh2* and a faster rate of improvement of cardiac function.

Initially two different doses of antagomir (20 mg/kg and 100 mg/kg) were tested and 20 mg/kg was chosen, as it was sufficient for inhibition of *let-7c*. A single injection of the *let-7c* antagomir at 20 mg/kg downregulated *let-7c* for several weeks in mice [37,38]. We found a single injection of *let-7c* antagomir to result in *let-7c* silencing in the cryoinjured fish in all organs tested; the heart, the liver, and the kidney. Variation of the qPCR results was highest in the heart samples, which may be partly explained by the very small size of the heart compared to that of the kidney and the liver. The variation of expression together with the small *n* = 3−5 resulted in not reaching statistical significance in all the comparisons. *let-7c* expression was at 7 dpi 21-fold, at 14 dpi 11-fold, and at 28 dpi 11-fold in the cryoinjured scramble hearts compared to the cryoinjured antagomir hearts, reaching statistical significance at 7 dpi, a time-point where cardiomyocyte proliferation and epicardial activation were found to be increased with *let-7c* silencing.

Fibrin deposition and scar formation after cryoinjury are essential for stimulating cardiomyocyte proliferation and cardiac remodeling in zebrafish [19]. In our study we observed decreased fibrin in the infarct area at 28 dpi after *let-7c* inhibition. We further looked at fibrinolysis and found that *pai-1* shows a trend of lower expression in *let-7c* antagomir fish hearts at 28 dpi. *pai-1* is the main inhibitor of plasmin, a serine protease, which dissolves many blood proteins including fibrin [39]. Adult zebrafish hearts completely regenerate after cryoinjury, unlike mammal hearts [4,5]. Injury stimulates expression of the developmental genes *raldh2, tbx18* and *tcf21* in the epicardium [13,15,16]. Activated epicardium is a hallmark of heart regeneration in adult zebrafish [13,14]. Hence, we quantified the expression of *raldh2*, an epicardial marker and found increased expression at 7 dpi in the *let-7c* antagomir fish hearts. Retinoic acid signaling plays an important developmental role in the embryonic zebrafish heart [40]. *raldh2* oxidizes retinaldehyde to retinoic acid and regulates tissue levels of retinoic acid. In the adult zebrafish heart, *raldh2* expression is upregulated after cryoinjury and inhibition of *raldh2* affects cardiomyocyte proliferation [13,14,15]. We examined cardiomyocyte proliferation by PCNA staining and found an increase in the number of proliferating cardiomyoctes in *let-7c* antagomir fish hearts at 7 dpi, in agreement with increased *raldh2* expression at 7 dpi. At later time-points cardiomyocyte proliferation was similar between *let-7c* antagomir and scramble fish. Our results are in agreement with previous studies, which have found similar amounts of PCNA positive cardiomyocytes in the heart after cryoinjury, with cardiomyocyte proliferation peaking during the first week after cryoinjury [5,41]. Additionally, we used BRDU baths to detect proliferating cardiomyocytes. However, we found that administration of BRDU by fish water to be inefficient in getting BRDU into the heart, as we observed a weak BRDU signal in stainings. After activation of epicardium, epithelial-mesenchymal transition gives rise to fibroblasts [13,14,15]. Fibroblasts are essential for regeneration; one study has reported that genetic ablation of fibroblasts negatively affects cardiac remodeling [19]. However, we found that expression of fibroblast associated genes *col12a1a* and *postna* to remain similar in *let-7c* antagomir and scramble fish hearts.

Echocardiography is the method-of-choice for studying cardiac function. However, due to the small size of the adult zebrafish (3–4 cm) and the very small size of the ventricle (750 μm), obtaining reproducible and reliable data is challenging [42]. The zebrafish ventricle is highly trabeculated, thus determination of the endocardial border is difficult [43]. We found that FVS measured from the epicardial borders of the ventricle is the most reliable method to quantify cardiac function in zebrafish. We found the recovery of cardiac function after cryoinjury essentially similar in *let-7c* antagomir and scramble fish. However, more proliferating cardiomyocytes in the *let-7c* antagomir fish at 7 dpi may reduce cardiac contractile function, as they are still too immature to contribute to it at this early time-point. Cardiomyocyte proliferation peaks at 7 dpi during heart regeneration in zebrafish, followed by restoration of myocardium and removal of fibrotic tissue at 14 dpi and later time-points [44,45]. Indeed, the recovery is statistically faster in fish receiving *let-7c* antagomir at 14 dpi (Figure 6).

Studies in mice show beneficial effects of silencing *let-7c* on cardiac recovery; one-study reports improved cardiac function with increased recruitment of epicardial cells [23]. Another study reports reduced fibrosis and improved cardiac function [30]. Consistently, we show *let-7c* inhibition to increase the number of proliferating cardiomyocytes, increase the expression of the epicardial cell marker *raldh2* and to accelerate the rate of recovery of cardiac function.

In conclusion, our study reports a favorable response to inhibition of *let-7c* in heart regeneration after cryoinjury in adult zebrafish. We show increased cardiomyocyte proliferation and epicardial activation at 7 dpi following inhibition of *let-7c*, as well as faster recovery of cardiac function and faster fibrinolysis. On the basis of these results, further studies on inhibition of *let-7c* to improve cardiac function are warranted.

## Figures and Tables

**Table 1:** Primer sequences.

Target	Primer Sequences	Source	Reference
Plasminogen activator inhibitor–1 (*pai-1)*	Forward 5′-GAGCGTCCCACACCAGATAG-3′ Reverse 5′-GCACTCCAGATGGGAGGAAC-3′	Sigma-Aldrich	This study
Collagen 12ala (*col12a1a)*	Forward 5′-GGTGAAAGAGGAGACACTGCGT-3′ Reverse 5′-AGTTGCTGGGGATCTGGTT-3′	Sigma-Aldrich	This study
Periostin (*postna)*	Forward 5′-CAAGGATCAAGACGAAGAGCAAG-3′ Reverse 5′-ATCTCAGGGTCTCCATTCATCT-3′	Sigma-Aldrich	This study
Retinaldehyde dehydrogenase 2 (*raldh2)*	Forward 5′-ACAGTGCTTACCTTGCTACCC-3′ Reverse 5′-CTTATCTGCCCATCCAGCGT-3′	Oligomer Oy	[35]
Ribosomal protein S3 (*rps3)*	Forward 5′-CGTGTCACACCAACAAGA-3′ Reverse 5′-CAGCTTGTAGCGCAGAGA-3′	Oligomer Oy	[35]
